# Orthopaedic Oncology in Contemporary Practice: A Comprehensive Review of Primary Bone Tumours, Metastatic Pathways, and Evolving Surgical Techniques

**DOI:** 10.7759/cureus.110438

**Published:** 2026-06-08

**Authors:** Parishekh Paulraj Jawahar, Ravinandan H A, Utkarsh Mahera, Parampreet Singh Mundh, Jassim Rahiman K, Ashish Kumar Shukla, Sukanta Bandyopadhyay

**Affiliations:** 1 Department of Orthopedics, Apollo Hospitals Old Mahabalipuram Road (OMR), Chennai, IND; 2 Department of Orthopedics, Mysore Medical College and Research Institute, Mysore, IND; 3 Department of Orthopedics, Rajiv Gandhi University of Health Sciences (RGUHS), Bengaluru, IND; 4 Department of Orthopedics, Shalby Hospital, Ahemdabad, IND; 5 Department of Orthopedics, Amandeep Hospital, Amritsar, IND; 6 Department of Kayachikitsa (Internal Medicine), All India Institute of Ayurveda, University of Delhi, New Delhi, IND; 7 Department of Radiodiagnosis, Santosh Medical College, Santosh Deemed to Be University, Ghaziabad, IND; 8 Department of Biochemistry, Rama Medical College Hospital and Research Centre, Kanpur, IND

**Keywords:** bone metastasis, limb salvage, musculoskeletal oncology, sarcoma, tumour reconstruction

## Abstract

Orthopaedic oncology encompasses the diagnosis and management of primary bone sarcomas and metastatic skeletal disease, requiring integration of oncologic principles with complex musculoskeletal reconstruction. Advances in molecular classification, cross-sectional imaging, systemic therapy, and surgical technology have substantially transformed contemporary clinical practice. This comprehensive review synthesises current evidence across epidemiology, tumour biology, diagnostic strategies, staging systems, multidisciplinary management, reconstructive techniques, complication profiles, and survivorship considerations in modern orthopaedic oncology. Contemporary classification frameworks incorporating molecular and genetic insights have improved diagnostic precision and prognostic stratification. Structured staging systems and validated risk assessment tools facilitate clinical decision-making, particularly in metastatic bone disease, where the prediction of pathological fracture and mechanical instability guides prophylactic intervention. Progress in limb-salvage surgery, modular endoprosthetic reconstruction, intercalary replacement, biologic reconstruction, and technology-assisted resection has expanded functional preservation while maintaining oncologic safety. Integration of radiotherapy and systemic therapies, including targeted and multimodal regimens, has enhanced survival in selected malignancies, shifting emphasis toward durable reconstruction and quality-of-life outcomes. Persistent challenges include tumour heterogeneity, infection and mechanical complications, and variability in long-term functional reporting. Contemporary orthopaedic oncology is defined by multidisciplinary coordination and biologically informed surgical strategy. Continued refinement of molecular stratification, standardised outcome assessment, and technological innovation remains essential to optimise oncologic control and functional recovery in patients with primary and metastatic bone tumours.

## Introduction and background

Orthopaedic oncology has become a highly specialised field that combines oncologic principles with complex musculoskeletal reconstruction for the management of primary bone sarcomas and metastatic skeletal disease [1]. Its central clinical goal is to achieve durable tumour control while preserving limb function, mobility, and quality of life whenever oncologically appropriate. Advances in multimodal treatment have led to significant improvements in survival in high-grade malignancies such as osteosarcoma, shifting clinical attention toward long-term survivorship, durable local control, functional preservation, and reconstruction durability [2]. Contemporary classification systems have improved histopathological classification and have matched diagnostic criteria with new molecular knowledge for better prognostic classification, as well as for therapeutic planning [3]. Modern staging schemes for bone and soft tissue sarcomas now incorporate both anatomical extent and biological behaviour, which helps reinforce systematic assessment in determining treatment algorithms [4]. Population-level analyses and national audits show the growing procedural complexity and centralisation of care in specialised units for musculoskeletal oncology, reflecting the need for multidisciplinary coordination [5]. Current controversies surrounding the role of optimum surgical margins, reconstruction options, and treatment of rare tumour subtypes highlight ongoing uncertainty in selected areas of clinical practice and the need for evidence-based consensus [6]. The development of fellowship-trained specialists in an organised manner has marked the maturation of the subspecialty of orthopaedic oncology [7]. Educational trends also point to an increasing amount of oncologic content in orthopaedic training curricula, in line with the increasing clinical load of skeletal tumours [8].

In addition to these core clinical developments, health-system and digital factors have become relevant supportive issues in contemporary orthopaedic oncology. The digital environment has brought new dimensions to patient education and the dissemination of information. There is still concern regarding the variable quality of information available in online orthopaedic oncology resources [9]. Economic analyses show a continued decline in reimbursement for oncologic surgical procedures, which creates financial pressures that could influence practice patterns as well as institutional resource allocation [10]. Longitudinal assessments of Medicare payments also show decreasing trends in compensation despite increasing technical complexity and operating room demands [11]. Parallel growth in machine learning-based prognostic modelling has led to interest in predictive analytics; however, methodological limitations and inconsistent adherence to open science standards continue to pose barriers to clinical translation [12]. Mobile health technologies and application-based tools have become adjuncts for diagnosis, operative management, and follow-up in orthopaedic oncology as part of the digital transformation of the surgical field [13]. Studies on initiatives in calcium and bone endocrinology highlight the biological complexity of skeletal homeostasis and its implications for tumour-bone interactions, strengthening the need to integrate oncology, endocrinology, and orthopaedics [14]. Perioperative optimisation strategies, such as transfusion management protocols, continue to influence outcomes in extensive oncologic resections requiring major reconstruction [15]. These topics are relevant to contemporary practice, but the introduction was revised to keep the main focus on diagnosis, staging, tumour control, reconstruction, and survivorship.

Consensus-building exercises in rare malignancies such as chondrosarcoma reveal priority areas for molecular and surgical techniques and highlight persistent evidence gaps [16]. Complication profiles specific to orthopaedic oncology, including increased risks of venous thromboembolism, require thromboprophylaxis strategies beyond routine orthopaedic practice [17]. Musculoskeletal infection is an important cause of morbidity after tumour resection and endoprosthetic reconstruction and requires integrated preventive and therapeutic strategies [18]. Developments characterise modern orthopaedic oncology as a multidisciplinary, technology-assisted speciality based on accurate staging, biologically informed diagnosis, careful surgical planning, and evolving systemic therapies. A practical way to understand current practice is to view each case through three linked questions: what is the biological behaviour of the tumour, what is the mechanical risk to the skeleton, and what functional outcome is realistically achievable for the patient? This framework connects tumour biology, fracture risk, reconstructive feasibility, and patient-centred outcomes, thereby moving clinical decision-making beyond technical treatment selection alone. The combination of molecular biology, advanced imaging, surgical innovation, and health-system factors continues to shape clinical practice. This review compiles current evidence in epidemiology, tumour biology, diagnostic strategies, staging, multidisciplinary treatment, and operative management, offering a comprehensive framework for contemporary practice.

Objective of the review

The purpose of this review is to present an overview of modern orthopaedic oncology practice, including epidemiology, molecular biology, diagnostic strategies, staging systems, and multidisciplinary management of primary and metastatic bone tumours. It is intended to bring together existing knowledge on surgical methods, reconstructive innovations, systemic treatments, complication management, and advances in risk stratification and functional outcomes. The review also aims to provide an integrated clinical perspective showing how biological classification, mechanical stability assessment, and reconstructive planning can be combined to guide decision-making in modern musculoskeletal oncology.

Methodology

This article was prepared as a comprehensive review of contemporary orthopaedic oncology. A literature search was conducted using PubMed, Google Scholar, Scopus, and Web of Science, with emphasis on peer-reviewed articles published from 2018 to 2025. Older studies were included when they provided established background information, staging systems, or foundational clinical concepts. Search terms included “orthopaedic oncology,” “bone tumours,” “primary bone sarcoma,” “metastatic bone disease,” “bone metastasis,” “limb salvage,” “endoprosthetic reconstruction,” “fracture risk,” “Mirel score,” “radiotherapy,” “systemic therapy,” and “musculoskeletal oncology.” Articles were included if they were published in English, appeared in peer-reviewed journals, and were relevant to primary bone tumours, metastatic skeletal disease, tumour biology, diagnosis, staging, treatment, reconstruction, complications, survivorship, or related health-system factors. Articles were excluded if they were non-English publications, conference abstracts without full text, duplicate publications, editorials or letters without sufficient clinical or scientific content, unrelated to orthopaedic oncology, or not supportive of the topics discussed. The selected literature was organised according to the main themes of the review. Evidence was synthesised descriptively across epidemiology, tumour biology, diagnostic workup, staging, multidisciplinary treatment, surgical reconstruction, complications, and future directions. This article was not designed as a systematic review; therefore, PRISMA-based study selection, formal risk-of-bias assessment, and meta-analysis were not performed. No formal statistical synthesis was performed because this article was designed as a comprehensive descriptive review rather than a quantitative evidence synthesis. Therefore, pooled effect estimates, confidence intervals, heterogeneity testing, meta-analysis, and meta-regression were not applicable. Since no advanced statistical analysis was performed, additional statistical peer review was not required.

## Review

Classification and epidemiology of bone tumours

Primary malignant tumours of the bone account for a small percentage of the total cancer incidence but disproportionately affect children, adolescents, and young adults and cause significant long-term morbidity in those who survive [19]. Histologic zones once thought ambiguous have become firmly established based on molecularly defined sarcoma subtypes and are firmly embedded in existing classifiers to satisfy the incorporation of genetic profiling [20].

Immune-Genomic Interactions in Synovial Sarcoma

A demonstration of the influence of tumour-specific transcriptomic programs on tumour biology and sensitivity to therapy [19]. Advances in molecular pathology have helped improve the reproducibility of the diagnosis and better prognostic differentiation of musculoskeletal malignancies [20]. The central role in sarcomagenesis is played by chromatin dysregulation and aberrant growth factor signalling, and invasion potential and uncontrolled proliferation are facilitated by the co-option of receptor tyrosine kinases [21,22]. Breaking of tumour suppressor mechanisms, such as NF2/merlin-associated signalling pathways, further characterises mechanisms of neoplastic transformation in connective tissues [21]. Metabolic reprogramming via transcriptional regulation of glycolytic pathways maintains tumour growth in hypoxic and nutrient-limited conditions, reinforcing the idea of cancer as a metabolic disease [23].

Secondary skeletal involvement due to systemic malignancies is one of the key causes of clinical burden in orthopaedic oncology practice. Chemokine-mediated signalling pathways control tumour cell homing to bone and support the biological basis of preferential metastasis of tumour cells to bone [24]. The chemokine receptor (CXCR4) axis and associated chemotactic gradients promote the migration towards bone marrow niches, where there is an interaction of the tumour cells with the stromal and immune components of the marrow to create metastatic colonies [25].

Chemokine-Induced Migration

Tumour cell migration further supports the role of cytoskeletal dynamics and intracellular signalling in metastatic dissemination [25]. Epidemiological patterns of metastatic disease of the bone are indicative of the frequency of primary malignancies, including breast, prostate, and lung carcinoma [26]. Osteolytic and osteoblastic lesions are opposing pathophysiologic processes caused by tumour-derived mediators and host response [23,27]. Recognition of these mechanisms informs the creation of classification systems that combine biological, radiologic, and clinical parameters [27]. Contemporary orthopaedic oncology is thus based on a synthesis of epidemiologic data, molecular taxonomy, and clinicopathologic staging as part of the therapeutic decision-making process and risk stratification [19].

Musculoskeletal oncology and tumorigenesis malignancy

Tumorigenesis in musculoskeletal oncology is a process involving an accumulation of genetic and epigenetic changes that transform cellular pathways of proliferation, differentiation, and survival [28]. Aberrant histone variants as well as growth factor receptor signalling have been implicated in the oncogenic process, implicating chromatin remodelling in oncogenesis [29]. NF2/merlin-associated molecular pathways are involved in the control of cytoskeletal organisation and contact inhibition, and disruption of these pathways promotes tumour progression in select neoplasms [30]. Metabolic adaptation is an imbalance of cancer biology at the most basic level. Transcriptional regulators of the Warburg effect are driving increased glycolytic flux even in the presence of oxygen, which leads to biosynthetic capacity and resistance to cellular stress [22]. These metabolic changes are involved in the development of aggressive phenotypes and potentially represent therapeutic targets of treatment in the management of sarcoma [12]. Modulation of the cluster of differentiation 164 and C-X-C motif CXCR4 (CD164-CXCR4) signalling axis affects tumour aggressiveness and metastatic competence in Ewing sarcoma, a link between chemokine biology and clinical behaviour [23].

**Figure 1:**
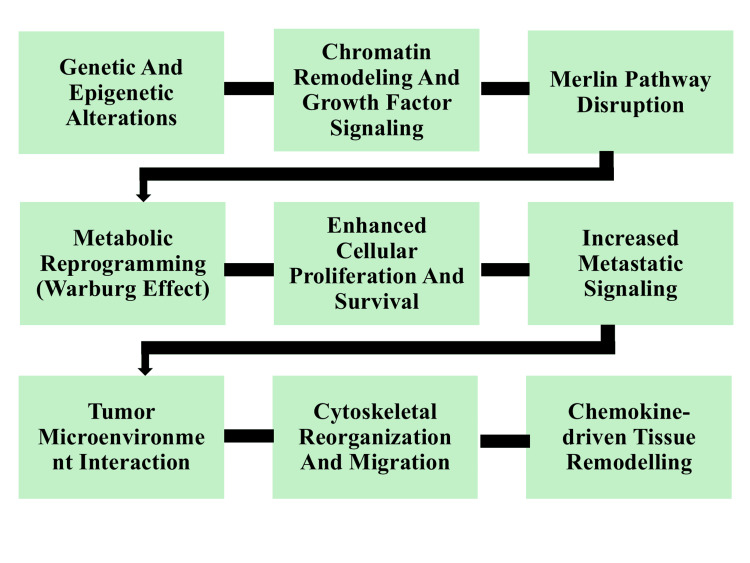
Molecular mechanisms of tumorigenesis in musculoskeletal oncology. Created by the authors using Microsoft PowerPoint (Microsoft Corporation, Redmond, WA, USA).

The tumour microenvironment contributes to musculoskeletal tumour progression by supporting invasion, immune modulation, angiogenesis, and metastatic spread [31]. Chemokine signalling, including CXCR4-related pathways, may influence tumour cell migration and interaction with the bone marrow niche [24,25]. These mechanisms are clinically relevant because they help explain patterns of skeletal metastasis, tumour-induced bone destruction, and tissue remodelling [27]. Molecular subtyping also has practical value by improving prognostication and identifying potentially actionable alterations for precision oncology strategies [19,32]. These developments reflect the shift from purely morphology-based diagnosis toward biologically informed classification systems that connect pathology, systemic therapy, and surgical planning [19]. The molecular pathways involved in musculoskeletal tumorigenesis are summarised in Figure 1.

Pathophysiology of metastatic bone disease

Metastatic bone disease is a complex series of biological events involving tumour cell detachment, vascular dissemination, extravasation, and colonisation of the bone marrow microenvironment [23]. CXCR4-ligand interactions, especially those between the ligand and receptor, CXCR4, are responsible for the directed homing of circulating tumour cells to skeletal niches [33]. Experimental studies of chemokine-induced migration have shown the central role of the intracellular signalling networks in the control of cytoskeletal reorganisation and motility [34]. Once established in bone, metastatic cells impair normal remodelling processes through activation of resorption mediated by osteoclast or stimulation of osteoblastic proliferation. These divergent pathways have osteolytic or osteoblastic lesions possessing different radiographic and biomechanical characteristics. The effects are enhanced by inflammatory cytokines and chemokines, which recruit part of the immune and stromal cells involved in the metastatic niche [35]. The chemokine signalling pathway represented by the CXCL1-CXCR2 axis is a good example of how chemokine signalling helps to modulate tissue remodelling and cellular infiltration, processes that are similar to metastatic bone destruction [36].

**Figure 2:**
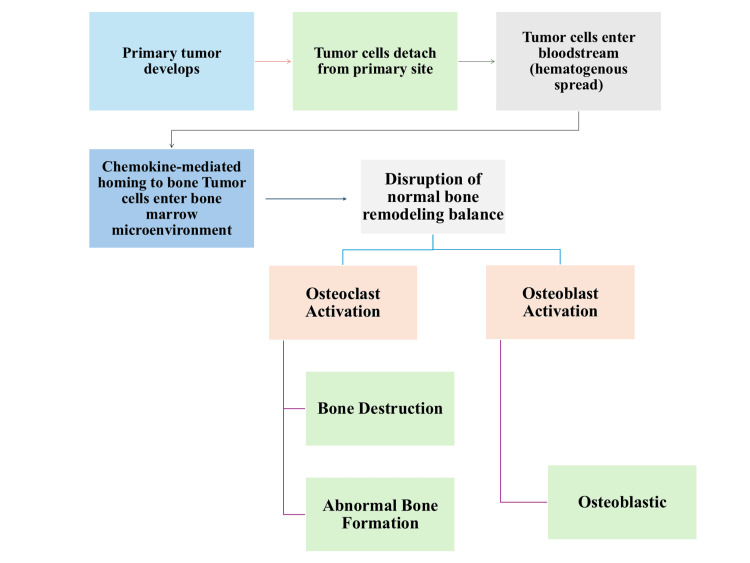
Pathophysiology of metastatic bone disease. Created by the authors using Microsoft PowerPoint (Microsoft Corporation, Redmond, WA, USA).

The structural implications of dysregulated remodelling are cortical thinning, trabecular compromise, and mechanical instability leading to pathological fracture and spinal cord compression [37]. The identification of molecular agents of skeletal colonisation can be applied to risk assessment plans and intervention treatments. Integration of the biology of chemokines with clinical scoring systems favours the early detection of high-risk lesions and facilitates the timely prophylactic stabilisation [38]. Metastatic bone disease, thus, is the coming together of a systemic malignancy with specific biological characteristics of the skeletal tissue. Two different aspects must be appreciated in order to become an effective manager: the molecular mechanism and the biomechanical implications [23,29]. This will allow for coordinated surgical and system-wide treatment planning [23]. The biologic sequence of metastatic bone disease is shown in Figure 2.

Clinical Presentation and Diagnostic Workup

Suspected bone tumours are a process of symptomatology, imaging results, and histopathologic confirmation [39]. Oncologic biopsy tenets require meticulous thinking about the position of the incisions and orientation of the tract to maintain subsequent resection possibilities and to reduce contamination of uninvolved compartments [28]. Image-guided core needle techniques offer a high yield of diagnosis with lesser procedural morbidity [28]. Radiography is the basis of diagnostic imaging, with advanced cross-sectional imaging identifying lesion extent and soft tissue involvement [40]. Differential diagnosis and staging are done on the integration of imaging characteristics with clinical context. Quantitative risk assessment tools are especially relevant in metastatic disease, in which the prediction of impending fracture is a factor in operative decision-making. Internal validation studies of the modified Mirel's scoring systems show that they provide better discrimination for pathological femoral fractures [29]. Refinements to the location component of Mirel's score have added to the predictive accuracy in proximal femoral lesions [30]. Alternative scoring frameworks have been investigated in multiple myeloma as a solution for shortcomings of conventional indices and for the optimal fracture risk prediction [31]. These tools make it easier to stratify in an objective way and help with prophylactic fixation decisions in an effort to prevent catastrophic structural failure.

**Figure 3:**
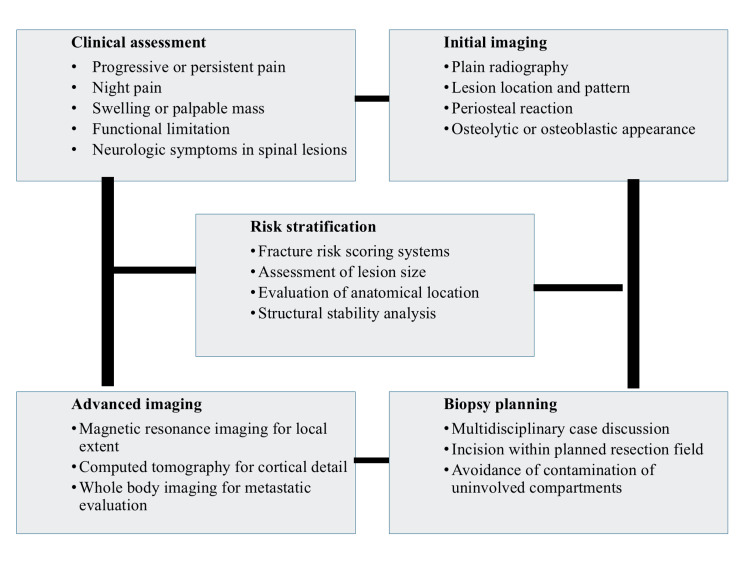
Diagnostic workflow in suspected bone tumours. Created by the authors using Microsoft PowerPoint (Microsoft Corporation, Redmond, WA, USA).

Histopathologic diagnosis involves morphologic examination using the enhancement of immunohistochemistry and molecular diagnostics consistent with modern systems of classification [3]. Properization of tissue diagnosis is still necessary in order to do therapeutic sequencing and prognostication [41]. A comprehensive assessment done by different specialists, like orthopaedic surgeons, radiologists, pathologists, and medical oncologists, can help reduce delays in the definitive management of the disease [42]. In orthopaedic oncology, direct clinical results in orthopaedic cancer partially require precise diagnosis of tumor characterization of tumor machinery, reconstruction planning, and selection of systemic therapy based on accurate diagnosis [28]. The step-by-step toggled diagnostic approach of suspected bone tumours is explained in Figure 3.

Staging and Prognostic Stratification

The orthopaedic oncology staging systems combine anatomical extent, histologic grade, and metastatic profile to provide information about therapeutic sequencing, supervision, and prognostic estimation [3,4]. Modern classification designs focus on congruence between radiology and biological behaviour and may be an entry point to the current transition from traditional staging designs towards consideration and incorporation of molecular calculations into traditional staging constructs [19].

Quantitative Tools to Predict Fractures: Adjunctive Information for the Prognosis of Metastatic Disease

Modified Mirel's scoring systems have been validated internally to enhance the reliability in predicting the risk for femoral fracture [29]. Adjustments to parameters of lesion location improve discriminatory capacity and avoid unnecessary operative intervention [30]. Novel scoring methods under testing in hematologic malignancies attempt to address limitations of current indices and improve the decision levels [31].

Prognostic stratification naturally includes progressively more molecular markers, such as lymphocyte CXCR4 expression patterns correlative to metastatic potential [43]. Knowledge of biologic factors that drive dissemination is in favour of risk indicator surveillance and therapeutic regimen. From a mechanistic basis, chemokine-mediated migration pathways also underscore the mechanistic basis for the progression of disease and may be determining for the design of future prognostic models [24]. Comprehensive staging thus moves beyond a description of anatomic characteristics to include biomechanical risk as well as molecular characterisation. Integration of these elements makes it possible to implement individualised treatment planning with a balance between the oncologic control and maintenance of function and quality of life [44]. Key staging and fracture risk assessment systems that are used in orthopaedic oncology are summarised in Table 1.

**Table 1:** Staging and fracture risk assessment systems in orthopaedic oncology. AJCC:  American Joint Committee on Cancer; TNM: Tumour, Node, Metastasis

System/Tool	Clinical Application	Core Components	Clinical Utility	References
Enneking Surgical Staging System	Primary bone tumors	Tumour grade, local extent, metastasis	Guides surgical margins and limb salvage decision-making	[4]
AJCC TNM Classification	Primary malignant bone tumors	Tumour size, nodal status, distant metastasis, and grade	Standardised oncologic staging and prognostic stratification	[3]
Mirel’s Scoring System	Metastatic long bone lesions	Site, pain, lesion type, size	Predicts risk of pathological fracture; guides prophylactic fixation	[29]
Modified Mirel’s System	Metastatic proximal femur lesions	Revised weighting of anatomical location	Improves fracture prediction accuracy	[30]
Novel Fracture Risk Models (e.g., Myeloma-specific systems)	Hematologic malignancy with bone involvement	Radiologic and structural parameters	Enhances prediction beyond traditional scoring	[31]

Multidisciplinary Management Strategies

Radiotherapy is part and parcel of the multidisciplinary treatment of a chosen primary and metastatic tumour of the bones. modulated radiotherapy and stereotactic body radiotherapy show that there are different toxicity profiles and therapeutic implications [32]. Stereotactic body radiotherapy delivers a small area of high-dose radiation while allowing some sparing of surrounding structures, and increasing the use for spinal and oligometastatic disease [33]. Evidence is available from several oncolytic scenarios to support its role as an alternative or adjunct with definitive surgery for proper patients [34]. Utilisation in complicated malignancies highlights the desirability of technologies and the shifting extreme radiotherapeutic models [35]. Cytotoxic chemotherapy and targeted agents are systemic therapies whereby surgical and radiotherapeutic strategies are coordinated in order to maximise disease control. Precision approaches to oncology based on molecular profiling optimise the choice of therapy and might help avoid overtreatment [45].

Integrating surgical stabilisation, systemic therapy, and radiation planning in specialised multidisciplinary teams is required for effective management [46]. Coordination between the orthopaedic surgeons, radiation oncologists, and medical oncologists helps in aligning the local and systemic treatment goals. Technological innovation continues to change the current patterns of practices, reinforcing the dynamic and collaborative kind of contemporary orthopaedic oncology [32]. Contemporary systemic and radiotherapeutic modalities of treatment are presented in Table 2.

**Table 2:** Contemporary systemic and radiotherapeutic treatment modalities.

Treatment Modality	Indication	Mechanism/Principle	Clinical Role	References
Conventional External Beam Radiotherapy	Primary or metastatic bone tumors	Fractionated ionising radiation	Local control, palliation of pain	[32]
Stereotactic Body Radiotherapy	Spinal or oligometastatic disease	High-dose conformal radiation	Precise tumour ablation with limited collateral damage	[34]
Chemotherapy (e.g., osteosarcoma protocols)	High-grade primary sarcoma	Cytotoxic systemic therapy	Improves survival and reduces recurrence	[2]
Targeted/Precision Therapy	Molecularly defined tumors	Pathway-specific inhibition	Personalised oncologic management	[19]
Bone-Modifying Agents (e.g., bisphosphonates, denosumab)	Metastatic bone disease	Inhibition of osteoclast-mediated resorption	Reduces skeletal-related events	[23]

Surgical Management in Contemporary Practice

Surgical resection is still the focus of curative treatment of primary bone sarcomas and of palliative treatment of metastatic lesions. Endoprosthetic reconstruction of the upper extremity allows limb saving while restoring the structural integrity and functionality [36]. Intercalary diaphyseal prostheses are durable segmental defect solutions and preserve the integrity of adjacent joints [37]. Advances in prosthetic design increase the reconstructive options in anatomically-reifying areas [38]. The robotic-assisted surgical platforms are emergent surgical adjuncts in oncologic resection, with the initial multicenter experiences supporting the feasibility and precision in complicated scenarios [39]. Comparative robots and open approaches point out the emerging change in the strategy of operations and possible decreases in the morbidity in the perioperative conditions [40].

The dilemma to choose limb salvage or amputation must be clearly evaluated on the oncologic margins, neovascularity, and the expected functionality. Systematic reviews in severe extremity trauma yield information that can be used in reconstructive oncologic surgery to assess risk versus benefit [41]. Meta-analytic data further help to understand decision-making and outcomes in limb-threatening conditions from a patient's perspective [43]. Surgical planning is done with the assistance of oncologic principles, with biomechanical reconstruction principles focusing on durable fixation and restoration of mobility. Advances in implant technology and operative technique are still allowing for an ever-increasing ability to salvage the limb with acceptable complication profiles [36]. Significant reconstructive methods and technological breakthroughs in surgical oncology are described in Table 3.

**Table 3:** Contemporary surgical and reconstructive strategies in orthopaedic oncology.

Surgical Strategy	Indication	Reconstruction Method	Advantages	References
Limb-Salvage Surgery	Resectable primary bone sarcoma	Wide excision with reconstruction	Preserves limb function; oncologic control	[36]
Endoprosthetic Reconstruction	Large segmental bone defects	Modular metallic prosthesis	Immediate stability and early mobilization	[36]
Intercalary Reconstruction	Diaphyseal tumor resection	Intercalary prosthesis or graft	Preserves adjacent joints	[37]
Robotic-Assisted Tumour Resection	Complex anatomical locations	Computer-guided precision resection	Improved accuracy and margin control	[40]
Amputation (selected cases)	Unresectable or neurovascular involvement	Definitive limb removal	Oncologic safety in advanced disease	[41]

Complications and Functional Outcomes

Orthopaedic oncology procedures have unique perioperative risks from massive dissection, immunosuppression, and complicated reconstruction. Venous thromboembolism is a major complication of surgery, and prophylactic measures are required in high-risk populations [17]. Musculoskeletal infection after tumour resection and implantation of endoprosthesis is one of the main causes of morbidity and implant failure [18]. Prophylactic antibiotic regimens for massive endoprostheses are in order to decrease the incidence of infections in high complexity reconstructions, continuing efforts to reduce postoperative complications [44]. Interventional radiology techniques help in the perioperative management, embolisation, and minimally invasive techniques complement surgical strategies [45].

The functional outcomes rely on the permanent reconstruction, prevention of infections, and proper rehabilitation. The distinction between spine instability secondary to tumour and degenerative pathology is still crucial to proper intervention planning [46]. Multidimensional postoperative surveillance incorporates the examination, imaging, and functional scoring mechanisms in determining long-term performance of implants and quality of life. Complication mitigation and functional optimisation are concurrently key goals in orthopaedic oncology, wherein survival advancements have led to a refocus on long-lasting reconstruction and long-lasting patient-reported outcomes [18].

Health Systems, Economics, and Quality of Care

Contemporary orthopaedic oncology practice is becoming more influenced by health system factors, reimbursement structures, and quality metrics that affect access to specialised care. National analyses of trends in reimbursement for surgical oncology procedures show continued declines in reimbursement for index cancer procedures despite increasing complexity of procedures and perioperative demands [10]. Longitudinal assessment of Medicare payments for two decades similarly shows progressive deflation of the payment for orthopaedic oncology operations, raising the concern about financial sustainability in high-resource surgical environments [11]. These economic pressures may impact how resources are allocated in the institution, workforce planning, and availability of specialised multidisciplinary services.

Workforce development in musculoskeletal oncology is indicative of structured subspecialization as fellowship leaders' analyses confirm the consolidation of expertise in academic centres [7]. Concentration of specialised training assists in high-volume practice models that have been linked to better oncologic and reconstructive outcomes. Educational trends also support growth in the content of oncology on orthopaedic training examinations, suggesting acknowledgement of management of tumours as a fundamental competency in the field [8]. Quality of care goes beyond operative technique to include perioperative optimisation, preventing complications, and evidence-based decision-making. Venous thromboembolism is a major topic of preventable morbidity in orthopaedic oncology patients, and standardised prophylactic protocols adapted to hypercoagulability associated with malignancy are required [17]. The use of infection prevention strategies for large-scale endoprosthetic replacement remains a topic of development, and prophylactic antibiotic guidelines are tested to reduce the occurrence of implant-associated morbidity among the high-risk groups [44].

Digital health tools and mobile applications have become part of the adjuncts for diagnosing this condition, monitoring of operations, and survivorship support, which is a bigger picture that involves integrating technology into the clinical path [13]. However, fluctuations in the quality of online information available to patients make it important that clinician-guided education and institutional oversight occur [9]. Machine learning-based oncology studies highlight the lack of transparency and reporting practices and highlight the importance of following the principles of open science to ensure reproducibility and clinical applicability [12]. Health systems infrastructure, economic sustainability, workforce development, and quality assurance are the combination of factors that affect the delivery of orthopaedic oncology care. Optimisation of these domains is integral to continuing equitable access and procedural excellence for the long-term patient outcome in an oncologic world of ever-increasing complexity [10].

Limitations and Future Directions

There are some limitations that primary bone tumours are clinically uncommon and biologically heterogeneous, which limits the possibility of conducting large randomised controlled trials and in many cases requires the use of retrospective or single-centre studies. Variability in staging systems, surgical techniques, and ways of reported outcome make it difficult to compare the results across published series. A feature of inconsistent long-term functional and survivorship reporting, also important in impeding a holistic understanding of long-lasting effects, is mapping the contexts of various reconstructive technologies and multimodal treatment approaches.

Future directions should focus on establishing multicenter collaboration in a structured way to produce more good-quality prospective data and a standardised form of reporting outcomes. Integration of molecular profiling into diagnostic and staging routines may result in improved personalised risk stratification and choice of therapy. Refinement of predictive tools for risk of fracture and disease progression to metastases will also be required to optimise the timing of surgery and use of resources. Continued technological innovation in imaging, navigation systems, implant design, and minimally invasive techniques is expected to make operations more precise and improve functional recovery. Greater attention to survivorship, patient-reported outcomes, and cost-effectiveness analysis will be critical in ensuring that advances in the field of oncology translate into sustainable and patient-centred orthopaedic oncology care.

## Conclusions

This review concludes that modern orthopaedic oncology is characterized by the integration of molecular biology, advanced diagnostic frameworks, and evolving surgical technologies to achieve optimal local oncologic control while preserving function. The discipline has shifted its confrontations to the amputation-dominated strategies to the limb-salvage model embraced by specific staging systems, bio-informed classification of data, and multidisciplinary treatment intentions. Improvements in systemic therapy and radiotherapy have led to improvements in survival in selected primary and metastatic bone malignancies, and therefore, the clinical focus is now more on durable reconstruction, prevention of complications, and long-term quality of life. Refinements in endoprosthetic reconstruction, intercalary techniques, and technology-assisted resection have increased surgical feasibility and have acceptable mechanical and infectious complications profiles. Quantitative risk stratification instruments have enhanced decision-making in metastatic disease, especially in the prevention of pathological fracture and structural instability. The future improvement should be based on the continued multidisciplinary teamwork, standardized outcome reporting, and the incorporation of accuracy oncology in the designing processes of surgery. Emphasis on survivorship, patient-reported outcomes, and cost-conscious care will continue to be central as the complexity of treatment continues to increase. Orthopaedic oncology is, thus, a dynamic and fast-evolving discipline in which scientific innovation and surgical excellence are combining to enhance outcomes.
